# Oral cancer screening practices of oral health professionals in Australia

**DOI:** 10.1186/s12903-017-0439-5

**Published:** 2017-12-15

**Authors:** Rodrigo Mariño, Satoru Haresaku, Roisin McGrath, Denise Bailey, Michael Mccullough, Ross Musolino, Boaz Kim, Alagesan Chinnassamy, Michael Morgan

**Affiliations:** 1 Oral Health Cooperative Research Centre, Melbourne Dental School, University of Melbourne, Melbourne, VIC 3010 Australia; 2 Department of Public Health & Human Sciences, Preventive & Public Health Dentistry, Fukuoka Dental College, Sawara-ku, Fukuoka, Japan; 3 Melbourne Dental School, University of Melbourne, Melbourne, Australia; 4 eviDent Foundation, South Yarra, Australia; 5 University of Melbourne, Melbourne, Australia

**Keywords:** Oral cancer, Screening practices, Self-confidence, Oral health professionals, Australia

## Abstract

**Background:**

To evaluate oral cancer-related screening practices of Oral Health Professionals (OHPs - dentists, dental hygienists, dental therapists, and oral health therapists) practising in Victoria, Australia.

**Methods:**

A 36-item survey was distributed to 3343 OHPs. Items included socio-demographic and work-related characteristics; self-assessed knowledge of oral cancer; perceived level of confidence in discussing oral health behaviors with patients; oral cancer screening practices; and self-evaluated need for additional training on screening procedures for oral cancer.

**Results:**

A total of 380 OHPs responded this survey, achieving an overall response rate of 9.4%. Forty-five were excluded from further analysis. Of these 335 OHP, 72% were dentists; (*n* = 241); either GDP or Dental Specialists; 13.7% (*n* = 46) were dental hygienists; 12.2% (*n* = 41) were oral health therapists, and the remaining 2.1% (*n* = 7) were dental therapists. While the majority (95.2%) agreed that oral cancer screening should be routinely performed, in actual practice around half (51.4%) screened all their patients. Another 12.8% “Very rarely” conducted screening examinations. The probability of routinely conducting an oral cancer screening was explored utilising Logistic Regression Analysis. Four variables remained statistically significant (*p* < 0.0001). Results indicate that the likelihood of conducting an oral cancer screening rose with increasing levels of OHPs’ confidence in oral cancer-related knowledge (OR = 1.35; 95% CI: 1.09–1.67) and with higher levels of confidence in discussing oral hygiene practices with patients (OR = 1.25; 95% CI: 1.03–1.52). Results also showed that dental specialists were less likely to perform oral cancer screening examinations compared with other OHPs (OR = 0.18; 95% CI: 0.07–0.52) and the likelihood of performing an oral cancer screening decreased when the “patient complained of a problem” (OR = 0.21; 95% CI: 0.10–0.44).

**Conclusion:**

Only half the study sample performed oral cancer screening examinations for all of their patients. This study provides evidence of the need for further oral cancer-related education and screening training for OHPs, which is vital to enhance oral cancer prevention and early detection.

## Background

Globally, cancers are the second most common cause of death with about one in every six deaths attributable to cancer [1]. In 2012, there were 14.1 million new cancer cases and 8.2 million cancer deaths worldwide [1]. Over the last few years, despite advances in early diagnosis and treatment, and in the survival rate, there has been an increase in the overall incidence of cancers [2]. The cost of cancer treatment has also increased with advancements in early diagnosis and treatment [2]. Among Head and Neck Cancers (HNC), the most commonly occurring are Oro-Pharyngeal Cancers (OPC), which include the lip, oral cavity, and oropharynx. Globally, OPC are the sixth most common malignancy [3]. Despite the technological advancement, cancer awareness and improvement in survival rates for some cancers [2], no significant improvement has been reported in the five-year survival rates for OPC [4, 5].

OPC is one of the few oral diseases encountered by the oral health team that has significant morbidity and premature mortality Tobacco products, alcohol consumption and sun exposure are the most recognized risk factors for OPC [6]. But the incidence of Human Papilloma Virus (HPV) related OPC has also been on the rise, particularly in younger adults who have never smoked or used other tobacco products [7, 8]. In Australia, HNC are the seventh most common cancers with an incidence rate of 17 cases per 100,000 persons [9]. In 2017, estimated deaths due to HNC is well over 1000 individuals (with three fourths of the reported deaths in males), that amounts to roughly 3.7% (*n* = 4956) of all new cancers diagnosed [9].

A recent effectiveness review on oral cancer screening, demonstrated conventional oral examination to be a feasible and satisfactory option for opportunistic screening in dental settings with sensitivity and specificity similar to breast and cervical cancer screening programs [4, 10–12]. Early diagnosis of OPC can greatly increase the five-year survival rates from 50% to more than 80% [6, 13].

OPC are known to be amenable to early detection as they primarily occur at sites that are accessible and visible during a non-invasive examination [4]. They are often preceded by a visible precancerous lesion enabling early detection and treatment [14]. The tumors’ proliferative factor and stage at the time of diagnosis largely determines the prognosis of cancers [15]. However, only 30% of OPC are identified at an early stage [16] with the majority (50%) being diagnosed at an advanced stage of metastasis (stage III or IV). This is largely due to late presentation, delayed diagnosis and lack of a clear referral pathway between doctors and dentists [17–21]. This forms the strongest argument for early diagnosis of OPC and initiating early treatment.

It is essential that OHPs such as dentists, dental hygienists (DHs), dental therapists (DTs), and oral health therapists (OHTs), understand the importance of conducting a thorough oral screening examination for malignant and potentially-malignant lesions as part of their routine clinical assessments, even in younger populations originally considered at lower risk for oral cancer [22, 23]. The World Dental Federation and Dental Associations, including the Australian Dental Association, proactively encourage Oral Health Professionals (OHP) to incorporate oral mucosal examinations as part of routine assessment [6, 24–27]. Several studies have assessed dentists’ knowledge, attitudes and practices regarding oral cancer [28–33]. However, only a few studies include DH, DT, and OHT. Thus, clinical screening practices regarding oral cancer among the complete range of oral health professionals remain largely unknown.

Screening programs should be designed to detect precancerous lesions or malignancies in their asymptomatic phases for all patients, not only in the older population groups, traditionally known to be at higher risk, but also in younger patient groups amongst which there has been a rising incidence of OPC [4, 7, 8, 34]. Nonetheless, studies among oral health professionals in Australia identified lack of training, lack of confidence and time constraints as the three most important barriers for oral cancer screening [4, 21]. Expansions of early diagnosis and treatment of oral cancers should be a priority from a public health viewpoint.

The purpose of this study was to investigate oral cancer screening practices of OHPs in Victoria, Australia as well as evaluate their oral cancer-related opinions and attitudes; and to identify factors associated with the likelihood of an OHP performing an oral cancer screening examination. This information can be used to identify gaps in oral cancer knowledge among OHPs, and subsequently to inform the development of continuing education programs specifically focused on oral cancer prevention, identification and management of malignant and potentially-malignant lesions of the oral cavity and oropharynx at the state level.

## Methods

The study was a cross-sectional survey of OHP in Victoria, Australia. As of September 2014, there were 4781 registered OHPs in Victoria, which amounts to 23% of the total Australian dental work force. Of them, 3715 are dentists and another 585 are registered DH/DT/OHTs in Victoria [35]. With the approval of the Human Research Ethics Committee at the University of Melbourne, a request was submitted to the professional associations representing Victorian OHPs, including the Australian Dental Association, the Dental Hygienists Association of Australia (Victorian Branch) and the Victorian Dental and Oral Health Therapists Association, to distribute the survey to their members. OHPs were initially contacted by mail in September 2014. Two weeks and four weeks after the first mailing, postcards were sent to thank those who had returned the questionnaire and to remind the others of the importance of the study, and encourage them to complete the survey. In an attempt to maximize the response rate, participants were offered a choice between completing either a paper-and-pencil survey or a web-based survey (i.e. invitation-letter with URL of online survey, questionnaire and reply paid envelope). Additionally, after the initial postal contact, the Australian Dental Association indicated a preference that e-mails be sent with the link to the online survey, as a reminder. Two reminders were sent in March and April 2015.

According to the literature [36–38] the expected response rate among OHPs is around 30%. However, an Australian study involving medical doctors, reported a mean response rate of 19.7% when using a similar simultaneous mixed-mode survey (a paper questionnaire and login details sent together) [39]. Therefore, we expected a final sample size of around 611 dentists and 89 dental hygienists/dental therapists/oral health therapists. The data collection period was from September 2014 to March 2015.

This sample size would be large enough to conduct the required statistical analyses as the minimum sample size necessary to study the relationship between a dependent variable (e.g., knowledge of risk factors for oral cancers) and a set of independent variables based on the case of 10 independent variables accounting for 5% of the variance (a conservative estimate) in the dependent variable, indicates that a sample size of 335 will yield a power of 0.80, that is, an 80% chance of explaining that proportion of the variance at a *p*-value of 0.05 [40].

Survey data was derived from a questionnaire consisting of four parts: socio-demographic and work characteristics (6 items); knowledge of risk factors for oral cancers (14 items); level of confidence in discussing health behaviors with patients (5 items); and oral cancer screening practices (11 items). The socio-demographic information included sex, age-range and location of practice. By occupational sub-stratification, participants were classified into three professional groups: ‘Dentists’; ‘Dental Hygienists (DH)’; and ‘Oral Health Therapists and Dental Therapists (OHT/DT)’. Dentists were further sub-divided into General Dental Practitioners (GDP) and ‘Dental Specialists’. Professional experience was classified into six categories: ‘5 years or less’; ‘6 to 10 years’; ‘11 to 15 years’; ‘16 to 20 years’; ‘21 to 25 years’; and ‘More than 25 years’. Employment history information included the postal code(s) of their main geographical location of practice. Using the Australia Post’s local delivery service guidelines, work locations were classified as being ‘Urban’, or ‘Rural’ [41].

Participants were asked to self-assess their level of knowledge about risk for oral cancer, utilizing a 10-point numerical scale (0 = ‘Very poor’ to 10 = ‘Excellent’). Knowledge regarding risk factors for oral cancer included 11 factors (smoking, periodontal disease, caffeine consumption, family history of oral cancer, chewing betel nut, human papillomavirus [HPV] infections, hepatitis C infection, chewing tobacco, level of alcohol consumption, herpes simplex virus infections and history of oral cancer). Participants were asked to select which of the 11 factors were risk factors for oral cancers utilising a three-option response of ‘Yes, ‘No’ or ‘Unsure’. To quantify the degree of knowledge of oral cancer risk factors among OHPs, a score system was developed, which involved adding the weighted answers for each of the 11 questions on risk factors. Scores ranged from 0 to 9. OHPs were also asked two questions regarding their perceived need for more training and education on oral cancers with response categories of ‘Yes’; ‘No’; or ‘Unsure’.

Questions related to oral cancer screening attitudes and practices asked: a) whether OHP should routinely screen for oral cancer; b) self-reported frequency of screenings (response options were: ‘Very rarely’; ‘With less than 50% of patients’; ‘With 50% or more of patients’; and ‘With every patient’); c) factors that prompt OHPs to initiate discussion of risk factors with patients; d) age groups routinely screened for oral cancer; e) whether OHPs inform the patient about being screened; f) if patients are informed what the screening involves; g) if they have ever used additional diagnostic tools to detect malignant lesions; and h) their course of action when an abnormal lesion was detected. Participants were also asked whether they had ever referred a patient to an oral medicine specialist.

Items focusing on OHPs’ level of confidence in discussing health behaviors with patients, utilized a numerical 10-point scale (0 = not at all confident to 10 = completely confident). These behaviors included; tobacco use, alcohol consumption, sexual behaviors, oral hygiene practices and diet and nutrition.

### Data analysis

The analysis provides descriptive information on the participants’ work and various socio-demographics. Bivariate associations were evaluated with Chi-squared analysis for nominal or ordinal variables. For variables on an interval scale, results were analyzed using one-way analysis of variance (ANOVA). A significant ANOVA was followed by post-hoc comparisons using Tukey’s Honestly Significant Differences tests. To better understand the association between the combination of socio-demographic, work and psychosocial variables and the probability of conducting a screening exam, a stepwise logistic regression analysis (LRA) was performed. All *p*-values <0.05 were considered significant. Data manipulation and analysis were conducted using IBM SPSS Statistics (Version 21.0, IBM Corporation, Endicott, NY, USA).

## Results

A total of 380 OHPs responded, achieving an overall response rate of 9.4% (ranging from 9.3% among dentists to 22.9% among DH/DT/OHTs). Fourteen respondents indicated that they do not practice dentistry anymore while another 31 mentioned that they do not routinely treat adult patients. These 45 OHPs were excluded from further analysis. Thus, a total of 335 were included in the final analysis. Most this group were dentists (72%; *n* = 241), either GDP (63.6%) or Dental Specialists (8.4%). Another 13.7% (*n* = 46) were DHs; 12.2% (*n* = 41) were OHTs, and the remaining 2.1% (*n* = 7) were DTs. By gender, the majority were female (58.2%). Among dentist participants, a marginally higher percentage were male (55.8%), while more than 90% of DH/DT/OHTs were female.

By age, more than half the total participants (55.5%) were 45 years or younger; however, the largest age group was the ‘46 to 55 years old’ group (24.8%), with 10.7% of the participants in the ‘25 years of age or younger’ group. Differences in age and gender were statistically significant by oral health profession (*p* < 0.001). Those working exclusively as OHT were younger than those working as dentists or DHs (See Table 1).

**Table 1:** Demographic, work characteristics of oral health professionals in Victoria

	Dentists	DHs	OHT/DTs	Total
	(*n* = 242)	(*n* = 45)	(*n* = 48)	(*n* = 335)
Age of group				*
25 or less	7.4	2.1	35.5	10.7
26–35	19.0	11.1	50.0	22.4
36–45	23.2	35.6	6.2	22.4
46–55	25.2	42.3	6.2	24.8
> 55	25.2	8.9	2.1	19.7
Gender				*
Male	55.8	2.2	8.3	41.8
Female	44.2	97.8	91.7	58.2
Duration of practicing				*
5 or less	15.7	11.1	80.5	23.0
6–10	12.4	11.1	17.1	12.8
11–15	11.6	13.3	2.4	11.0
16–20	9.9	15.6	0	9.9
21–25	9.9	20.0	0.0	9.9
> 25	40.5	28.9	0.0	33.4
Location of workplace				
Urban	76.6	77.3	74.5	76.4
Rural	23.4	22.7	25.5	23.6

*Chi- squared test; *p*-value: 0.001

When participants were asked about the location of their workplace, the majority (76.4%) indicated an ‘Urban’ location, with no statistically significant difference by professional background. Regarding the length of time practicing as an OHP, 33.4% indicated more than 25 years of practice; 30.8% between 11 and 25 years; 23.0% reported five years or less of practice; and the remaining 12.8% reported between 6 and 10 years of practice. As expected, differences by duration of practice between groups were statistically significant (*P* < 0.001).

Oral cancer screening examination.

Almost all OHPs (95.2%) indicated the importance of and need for routine oral cancer screening in their clinical practice. However, only half of those surveyed (51.3%) reported conducting a comprehensive oral cancer screening for all of their patients (Table 2). Another 21.8% reported conducting a comprehensive oral cancer screening for ‘*50% or more*’ of their patients. OHPs who reported performing oral cancer screening in ‘*less than 50% of patients*’ or ‘*Very Rarely’* comprised 14.0% and 12.8% of respondents, respectively. Differences by professional background for completing oral health screening were not statistically significant. The vast majority of respondents (92.2%) routinely screened patients 40 years of age or older for oral cancer. This proportion was 68.4% for 20–39 years old patients, and 38.5% for patients younger than 20 years of age.

**Table 2:** Oral screening behaviors of oral health professionals in Victoria

	Dentists	DHs	OHT/DTs	Total
	(*n* = 242)	(*n* = 45)	(*n* = 48)	(*n* = 335)
How frequently do you complete a comprehensive oral cancer screening?				
With every patient	51.7	46.7	54.2	51.4
With 50% or more of patients	21.9	22.2	20.8	21.8
With less than 50% of patients	13.2	17.8	14.6	14.0
Very rarely	13.2	13.3	10.4	12.8
How often do you discuss risk factors for oral cancer with your patients?				
With every patient	5.8	8.9	8.4	6.6
With 50% or more of patients	22.7	40.0	33.3	26.6
With less than 50% of patients	47.9	35.5	41.7	45.3
Very rarely	23.6	15.6	16.6	21.5
What factors influence your decision to perform an oral cancer screening examination? (Multiple answers)				
Patient complains of a problem	32.2	31.1	41.4	33.6
Age of the patient	31.4	25.2	43.8	32.4
Medical history	26.9	31.1	43.8	29.9
Other	16.5	26.6	25.0	19.1

Among those OHPs who reported not conducting a cancer screening for all their patients (*n* = 193), the decision to screen was influenced by the patient complaining of an oral health problem (65.6%); the patient’s age (63.8%); or the patient’s medical history (53.3%). Another 35.0% of respondents indicated other factors influencing their decision to conduct an oral cancer screening examination, such as exposure to risk factors (i.e. alcohol consumption and smoking). Of note, only the ‘age of the patient’ as an influencing variable reached statistical significance (*p* < 0.05).

When excluding those who ‘Very rarely’ conducted an oral cancer screening examination on their patients, just over half of respondents (51.9%) indicated that they ‘Sometimes’ informed the patient that they were screening for oral cancer lesions. Another 19.9% ‘Always’ informed patients that they were screening for potentially malignant or malignant lesions. More importantly, almost one third (28.2%) of the OHPs, ‘Never’ informed patients that such screening assessments were taking place. No statistically significant differences were found by professional background.

After excluding those who reported ‘Very rarely’ conducting oral cancer screenings, for almost all OHPs (97.6%) the examination involved a visual inspection of the patient’s oral cavity and 81.1% also included an extra-oral visual inspection (Table 3). Meanwhile, a screening examination that “Always” included either a visual inspection of the oropharynx or conducting neck palpation was reported at lower rates (52.3% and 28.1, respectively).

**Table 3:** Oral cancer screening examination behaviors of oral health professionals who performed screening more frequently than “Very rarely”

	Dentists	DHs	OHT/DTs	Total
	(*n* = 210)	(*n* = 39)	(*n* = 43)	(*n* = 292)
Screening examination involved: Extra-oral visual inspection of the oral cavity				
Always	80.4	82.1	83.7	81.1
Sometimes	2.9	5.1	0.0	2.7
Never	16.7	12.8	16.3	16.2
A visual inspection of the oral cavity				
Always	98.1	93.0	100	97.6
Sometimes	1.4	7.0	0.0	2.1
Never	0.5	0.0	0.0	0.3
A visual inspection of the oropharynx				
Always	50.5	64.1	50.0	52.3
Sometimes	40.3	35.9	42.9	40.0
Never	9.2	0.0	7.1	7.7
Neck palpation				
Always	25.5	23.7	44.2	* 28.1
Sometimes	56.9	52.6	32.5	52.6
Never	17.6	33.7	21.3	19.3

*Chi- squared test; *p*-value: 0.05

Apart from neck palpation, which was significantly more frequently conducted among OHTs than other professional groups (*p* < 0.05), none of the other screening techniques reached statistical significance levels by OHP background. Thirty-three respondents (11.3%), most of whom were dentists, indicated using additional diagnostic aids/tools to identify potentially malignant lesions. The most commonly utilized additional diagnostic tools included biopsies and radiographs.

When asked if respondents had ever referred a patient to an oral medicine specialist to have a lesion investigated, which subsequently was diagnosed as a malignancy, 54.2% of respondents had either never encountered such a clinical scenario, or were unsure. Of those who had referred a patient (*n* = 136), the most common intraoral site of the malignant lesion (*n* = 41) was cancer of the tongue, followed by cancer of the floor of the mouth (*n* = 22); hard palate (*n* = 18); and the lip (*n* = 14). There were also eleven cases of bone cancer.

OHP respondents were asked about their clinical decision pathway once an abnormal lesion was detected and the practitioner could not reach a diagnosis. The most commonly reported courses of action included: ‘Referral to an oral medicine specialist’ (85.7%); ‘Follow-up at a later appointment’ (53.7%); and ‘Consultation with another colleague’ (45.4%). Least mentioned courses of action included: ‘Ask the patient to monitor the lesion’ (38.2%); and ‘Referral to the patient’s general medical practitioner’ (14.3%). Differences by professional background were statistically significant for: ‘Consultation with another colleague’ (*p* < 0.001), where DHs and OHT/DTs were more likely to pursue this course of action; and ‘Asking the patient to monitor the lesion’, whereby OHT/DTs (55.8%) were more likely to ask the patient to monitor the lesion compared with 35.9% and 35.9% for dentists and DHs, respectively (*p* < 0.05).

Regarding knowledge of oral cancer risk factors, 99.4% of participants identified smoking as a risk factor for oral cancer. The second most frequently identified risk factor was chewing betel nut and chewing tobacco products (98.2%). A history of oral cancer was the third most frequently identified risk factor (97.0%), followed by family history of oral cancer (96.1%), and alcohol consumption (94.6%). The oral cancer risk knowledge score ranged from 0 to 88 with a mean of 68.5 (s.d. 10.1).

When OHPs were asked how often they discussed risk factors for oral cancer with their patients, the largest group (45.3%) indicated discussing risk factors for oral cancer with less than 50% of their patients (Table 2). Another 21.5% reported they ‘Very rarely’ discussed risks factors with their patients. Discussion of risk factors in ‘More than 50% of patients’ or ‘With every patient’ was reported by 26.6% and 6.6% of OHP respondents, respectively. No significant differences were demonstrated by professional background.

Concerning participants’ level of confidence in discussing behavioral issues that may affect oral health with their patients, participants generally felt confident discussing oral hygiene practices (Mean 8.9; s.d. 1.6), diet and nutrition (Mean 8.3; s.d. 1.7), tobacco use (Mean 8.2; s.d. 1.8), and to some extent alcohol consumption (Mean 7.3; s.d. 2.2). In contrast, the mean level of confidence in discussing sexual behaviors (i.e. oral sex practices) was comparatively low at 3.6 (s.d. 1.8).

The probability of conducting an oral health screening examination (“Very rarely”: 0 vs. Other categories [i.e., ‘With every patient’; ‘With 50% or more of patients’; and ‘With less than 50% of patients’]: 1) was explored utilizing LRA with age, sex, profession, rurality and number of years of work experience, risk factor knowledge index, self-assessed oral cancer knowledge, and self-confidence items as independent variables (Table 4). After controlling for the other independent variables included in the model, four variables remained statistically significant [χ^2^(4) = 40.689; *p* < 0.0001]. Results indicated that dental specialists, were less likely to perform an oral cancer screening examination (OR = 0.18; 95% CI 0.07 to 0.52). As the level of self-assessed knowledge of oral cancer increased, so too did the likelihood of performing an oral cancer screening examination (OR = 1.35; 95% CI 1.09 to 1.67). Additionally, when the OHP’s level of confidence in discussing oral hygiene with the patient increased, the likelihood of conducting an oral cancer screening examination also increased (OR = 1.25; 95% CI 1.03 to 1.52). On the other hand, when the ‘Patient complained of a problem’ the likelihood of performing an oral cancer screening examination decreased (OR = 0.21; 95% CI 0.10 to 0.44). The variance for oral cancer screening examination, using the full model, was 22.5% (Nagelkerke ^2^r = 0.225).

**Table 4:** Regression coefficient, odds ratios and 95% confidence interval for odds ratios for the factors associated with the probability of conducting an oral health screening examination among oral health professionals in Victoria, Australia

	β coefficient	Odds ratio	95% Confidence interval
Dental specialist	−1.70	0.18	0.07 to 0.52
Self-assessed knowledge	0.30	1.35	1.09 to 1.67
Level of confidence (oral hygiene)	0.22	1.25	1.03 to 1.52
Patient complained of a problem	−1.56	0.21	0.10 to 0.44
Constant	−1.04		
Nagelkerke r^2^ = 0.225			

## Discussion

The present study represents an attempt to explore oral cancer-related practices and attitudes among different OHPs working in the Australian state of Victoria. This study’s results show little over 50% of OHPs perform comprehensive oral cancer screening with all patients. Although these results are not directly comparable due to wording of questions and response categories, they would contrast with earlier studies done in Australia where OHPs routinely performed oral mucosal screening on 85–95% of their recall and new patients [4, 21]. In the same manner, studies done elsewhere, reported that 85–89% of dentists and 66–78% of dental hygienists performed oral cancer examination for their patients, although they did not indicate frequency of performing these examinations [29, 30]. Apart from this, the present study is largely in line with one other previous study done among DH/DT and OHTs in Australia on the importance and need for routine oral cancer screening [33].

A high proportion indicated that they did so in almost all patients older than 40 years. However, only about two-thirds of respondents routinely screened patients 20–39 years of age. The study also indicated that OHPs, who screened all patients, were less likely to always include neck palpation, and to a lesser extent, visual inspection of the oropharynx in their oral cancer screening practice. This may be due to lack of confidence in undertaking a head and neck examination as evidenced in other study done in Australia [21, 33]. These findings are cause for some concern as many oral cancers may not present with visibly detectable signs or symptoms during the pre-malignant or localized stages, which represents a clinical window in which these lesions are most amenable to treatment [29]. Such early stage lesions could otherwise be detected by a comprehensive and thorough visual examination of high risk sites [22, 42] and facilitating patient education about oral cancer.

Furthermore, a comprehensive oral cancer screening examination would seem essential, particularly given the growing incidence of HPV positive oropharyngeal cancers [4, 22, 42, 43]. Consequently, it is important for OHPs to maintain and develop competence and confidence in neck palpation techniques and visual examination of the oropharynx as part of their oral cancer screening practices [22]. Where necessary, appropriate education programs should be developed to address the knowledge and skill gaps in this area [4, 21]. The present study showed that OHTs performed oral cancer screening somewhat more frequently than other OHP groups.

As the incidence of oral cancer continues to rise [9], the role of oral health professionals in the prevention, early identification and management of oral malignancies will become increasingly relevant to public health. In order to reduce the morbidity and mortality of oral cancer, it is necessary to implement programs aimed at prevention, early identification and diagnosis. Early identification would involve facilitating patient recognition of signs and symptoms and providing education about when to consult a health professional. Patient delays in seeking care could be reduced by self-examination for early clinical features and by population level educational interventions, particularly targeting those in the higher risk groups for oral cancer.

In recent years, awareness of cancer risk factors has increased due to wider media coverage of the issue [44]. However, studies indicate that in contrast to other types of cancers, the majority of the population are ignorant about oral cancer [45, 46]. The public may have a tendency to interpret oral cancer symptoms and signs as relatively minor, as problems that are likely to resolve on their own. Given the lack of general public understanding of oral cancers and inability to recognise clinical symptoms and signs [22], it can be inferred that patient presentation to an OHP regarding an oral lesion may be either absent or too late, which would adversely impact on early detection and treatment outcomes. In addition to these issues, some cancers may be asymptomatic and the absence of notable symptoms may further contribute to late diagnosis.

For these reasons, opportunistic oral cancer screening examinations conducted by OHPs remain an important mode for early identification and diagnosis. Results from the multivariate analysis indicate that after controlling for other variables, dental specialists tend to perform less oral screening compared with other OHPs. It is possible that dental specialists rely on GDPs to conduct comprehensive dental examinations while they focus solely on the condition for which the patient was referred them; however, this cannot be stated categorically as the relevant information was not collected in the survey. The odds of conducting an oral screening examination also decreased when the patient complained of oral pain. It could be inferred, although once again respondents were not asked about this in the survey, that OHPs addressed the presenting complaint on these occasions (e.g. pain) and did not conduct a more comprehensive oral examination. Unsurprisingly, the data indicated that OHPs with a higher self-evaluated confidence level in oral cancer-related knowledge had greater confidence advising patients and were more likely to perform oral cancer screening examinations [21]. The ultimate goal would be to have the whole oral health team collaborate with an integrated approach to oral health care, particularly in the context of oral cancer knowledge, screening practices and patient and community education.

When considering these results, certain limitations must be acknowledged. The low response rate may have introduced non-response bias, and combined with the self-reported nature of the data means it is not possible to generalize findings to all OHPs working in Victoria, Australia. Furthermore, response rates to online surveys have been demonstrated to be less than for surveys administered using a paper-based approach [47, 48]. However, the response rate is also related to the size of the population under study with larger populations requiring smaller response rates [48]. Thus, although the number of responses received was not as high as was hoped, the achieved overall response rate was within the expected range for online surveys among oral health professionals (2.5% to 26%) [49, 50].

Despite some minor differences, this study group is largely representative of the Victorian OHP population in terms of age, profession, gender and distribution. There was an overrepresentation of DH/DT/OHTs in the study sample, who make up 28% of total respondents but only 17% of all registered dental practitioners in Victoria [35]. When considering the socio-demographic profile, there is no available data to determine the age range of each dental practitioner group in the wider profession in the state of Victoria. However, the age profile of survey respondents (55.5% aged 45 years or younger) is very similar that for all dental practitioners in Australia at the time of the survey (56.7% aged less than 45 years old) [35]. The proportion of female dentists in the study also mirrored figures reported for the state of Victoria (57%) in 2015. Most dentists worked in urban areas (80.0%) [51].

## Conclusions

Together with risk behavior change, oral cancer screening has been shown to contribute to reductions in oral cancer mortality rates among high-risk individuals [22, 52]. It is highly probable that OHPs will see patients with oral cancer and many more with potentially malignant lesions in their professional lives [4, 21]. Present results suggest that continued efforts to enhance the quality and consistency of oral cancer screening practices are required, which should include education and training of OHPs in up-to-date and evidence-based screening methods. The recommendation to see an oral health professional at least once a year would only be beneficial if patients are routinely screening for oral cancer. The results from this study may assist in the development of oral cancer-related education and training programs for OHPs. Increased emphasis on regular training in oral cancer screening protocols and in patient education and counselling practices are imperative to improve oral cancer prevention and rates of early detection, to reduce the mortality-burden resulting from oral cancers.

## Abbreviations

DHs: Dental Hygienists
DT: Dental Therapists
GDP: General Dental Practitioners
HNC: Head and Neck Cancer
HPV: Human Papilloma Virus
OHP: Oral Health Professional
OHTs: Oral Health Therapists
OPC: Oro-Pharyngeal Cancers
